# Aging Related Transcriptomic Changes in the Mouse Models of Alzheimer’s Disease

**DOI:** 10.1093/geroni/igaa057.385

**Published:** 2020-12-16

**Authors:** Asli Uyar, Ravi Pandey, Christoph Preuss, Kevin Kotredes, Gareth Howell, Michael Sasner, Gregory Carter

**Affiliations:** 1 The Jackson Laboratory, Farmington, Connecticut, United States; 2 The Jackson Laboratory, Bar Harbor, Maine, United States

## Abstract

Alzheimer’s Disease (AD) is characterized by multiple clinical phenotypes and molecular signatures at different stages of the disease and aging is the major risk factor for sporadic AD. Aging and AD are linked at molecular, cellular and systems level with commonalities in inflammation and associated immune response in the brain. Mouse models of AD were developed that mimic various aspects of aging-associated neurodegeneration and inflammation. Research in mouse models of AD showed that drugs and treatments designed for AD can decelerate aging phenotypes suggesting efficient utilization of these models in aging research. We analyzed RNA-Seq transcriptomic data from transgenic mouse models of familial AD (APP/PS1 and 5XFAD) and knock-in mouse models of late-onset AD (APOE and TREM2) at the ages between 4-months and 24-months. The number of differentially expressed genes between transgenic/knock-in and WT mice increased by age in all mouse models. Gene set enrichment analysis identified metabolic pathways, including oxidative phosphorylation, altered in an age and genotype related manner in the brain of APP/PS1 and 5XFAD mice that recapitulate major features of amyloid pathology. Immunity related pathways were enriched in APOE4 model carrying Trem2*R47H mutation at >12 months-old. We also mapped the transcriptional signatures to co-expression gene modules of human LOAD from the AMP-AD consortium and observed correlations specific to each mouse model. Our study provides a detailed view of how the aging interacts with AD-relevant pathologies at the transcriptome level and demonstrates potential translational relevance of the AD mouse models in the context of human aging.

